# Effective contact and outcome after pulmonary vein isolation in novel circular multi-electrode atrial fibrillation ablation

**DOI:** 10.1007/s12471-016-0907-6

**Published:** 2016-10-17

**Authors:** P. Gal, T. J. Buist, J. J. J. Smit, A. Adiyaman, A. R. Ramdat Misier, P. P. H. M. Delnoy, A. Elvan

**Affiliations:** Department of Cardiology, Isala Klinieken, Zwolle, the Netherlands

**Keywords:** Atrial fibrillation, Ablation, Multi-electrode ablation, Effective energy, PVAC Gold

## Abstract

**Introduction:**

Pulmonary vein (PV) reconnection is frequently the cause of recurrence of atrial fibrillation (AF) after ablation. The second-generation gold multi-electrode ablation (Gold-MEA) catheter has a new design possibly resulting in improved lesion formation compared with its predecessor. We aimed to determine the association between effective radiofrequency applications with the Gold-MEA catheter and outcome after AF ablation.

**Methods:**

50 consecutive patients with paroxysmal AF underwent Gold-MEA (PVAC GOLD^TM^, Medtronic Inc.) ablation. The Gold-MEA catheter was navigated to the PV ostium by fluoroscopy. Duty-cycled radiofrequency ablations were performed at all PV ostia. Lesions were considered transmural when electrode temperature was >50 °C and power >3 W for >30 seconds. After the ablation procedure, patients visited the outpatient clinic at 3‑month intervals including 24-hour Holter ECGs.

**Results:**

Mean age was 56 years. All PVs were acutely isolated with the Gold-MEA catheter. Procedure time was 111 ± 22 minutes, ablation time was 24 ± 6.7 minutes and fluoroscopy time was 20 ± 8.1 minutes. No procedure-related complications were observed. One year after ablation, 60 % of patients were still free of arrhythmia recurrences after a single PV isolation attempt. The number of transmural lesions was associated with arrhythmia-free survival: 25.0 % in <72 transmural lesions, 64.3 % in 72–108 transmural lesions and 71.4 % in >108 transmural lesions (*p* = 0.029).

**Conclusion:**

PV isolation can be performed successfully with the Gold-MEA catheter, with a favourable safety profile. Transmurality of lesions was associated with ablation success and may improve AF ablation success.

## Introduction

Pulmonary vein isolation (PVI) has become an important treatment modality for symptomatic atrial fibrillation (AF) [1, 2]. The dominant triggers for paroxysmal AF come from the pulmonary veins (PVs). During PVI, a circumferential lesion set is created at the base of the PVs so that these PVs are electrically isolated from the left atrium [1, 2]. Several techniques are used to perform the ablation, including point-by-point radiofrequency (RF) catheter ablation, [3, 4] cryoballoon ablation, [5] laser balloon ablation [5–8] and multi-electrode ablation (MEA) [3, 9, 10]. The last-mentioned is no longer in use due to its association with asymptomatic cerebral embolism, [11–14] but the newly designed gold multi-electrode ablation (Gold-MEA) catheter, building on its predecessor, combines several characteristics that may improve lesion formation and reduce complications. Although PVI has proven to be an effective treatment for AF, some patients develop recurrences [3, 15]. Electrical reconnection of the PVs is considered the most important mechanism for AF recurrence [2]. In contrast to other ablation techniques, there is no surrogate marker for transmurality of lesions for the Gold-MEA catheter. We aimed to report the ablation characteristics of 50 patients, determine transmurality of lesions with the Gold-MEA catheter and describe its association with ablation outcome.

## Methods

### Patient population

Fifty consecutive patients with symptomatic paroxysmal AF who were accepted for primo PVI in our centre were included in this study. Data were collected in a prospective hospital database. All patients consented to their data being registered and used for publication, as did the Board of Hospital Administrators.

### Preablation protocol

All patients underwent a CT scan, to assess left atrial and pulmonary vein (PV) anatomy. Patients with common PV ostia, both left and right, were excluded from this analysis, as well as patients with accessory PVs. Patients were admitted 24 hours before the ablation procedure. During hospitalisation, the cardiac rhythm was continuously monitored in all patients. Transthoracic echocardiography was performed routinely 1 day before ablation to determine right and left ventricular function, valvular abnormalities, and left and right atrial dimensions. Transoesophageal echocardiography was performed directly pre-ablation to assess the interatrial septum and to rule out intracardiac thrombus and/or severe aortic plaques. Routine blood tests were performed, including electrolytes and cardiac enzymes. Patients who used oral anticoagulants were ‘bridged’ using low-molecular-weight heparins up to 3 days prior to the ablation procedure, in accordance with local guidelines.

### Ablation protocol

All ablation procedures were performed under general anaesthesia supervised by a cardiovascular anaesthesiologist. After placement of a 6F quadripolar catheter in the coronary sinus via a transfemoral approach, a single transseptal puncture was performed using a Brockenbrough needle under fluoroscopic and pressure guidance. Immediately after the transseptal puncture, 10,000 IU of unfractionated heparin was administered. An 8.5F sheath (SL-1, St. Jude Medical, Minnetonka, MN, USA) was used for PV angiography. All sheaths were continuously flushed with saline containing 2500 IU heparin per 500 ml saline. An activated clotting time between 300 and 350 seconds was targeted. Additional heparin was administered when needed. The activated clotting time was checked during the procedure at regular intervals of 30 minutes.

### Multi-electrode catheter ablation

**Fig. 1:**
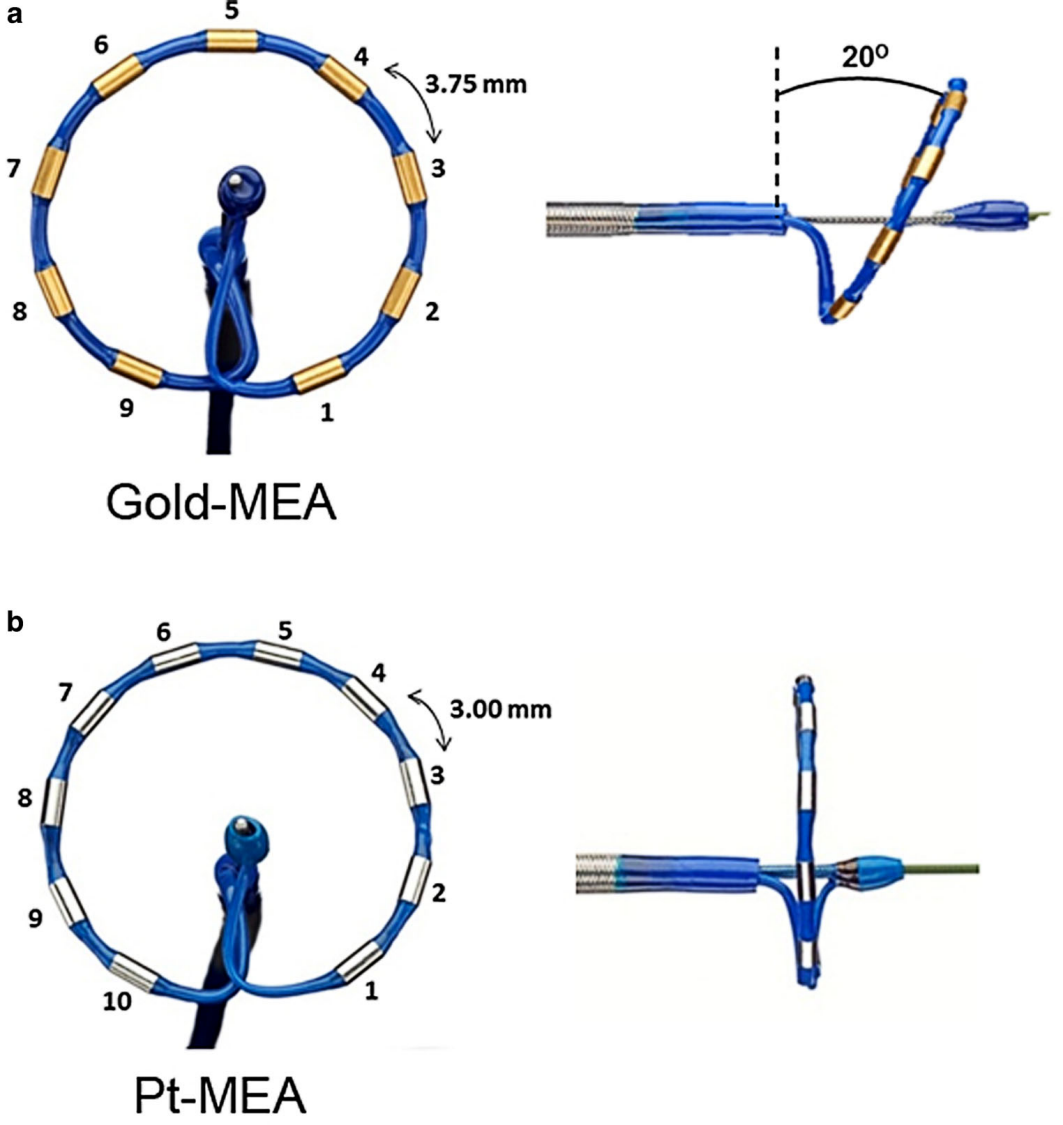
PVAC Gold Design. **a** Displays the newly designed Gold-MEA catheter; **b** displays the Platinum-MEA catheter. Several key improvements have been implemented: the ablation electrodes are made of Gold (Au) instead of Platinum (Pt); the new catheter consists of 9 electrodes, to prevent overlap of the 1^st^ and 10^th^ ablation electrode; the inter-electrode spacing is increased to 3.75 mm to retain the effective arc length and finally the electrodes are at a 20º forward tilt. *MEA* multi-electrode ablation

The multi-electrode pulmonary vein RF ablation catheter (PVAC Gold^TM^, Medtronic, Minneapolis, MN, USA) is a mapping and ablation catheter with a 25 mm diameter circular electrode array. This catheter has a bidirectional steering mechanism and an over-the-wire design. Compared with its predecessor, the novel Gold-MEA catheter consists of 9 gold electrodes positioned with 3.75 mm inter-electrode spacing at a 20° forward tilt for optimal electrode-tissue energy transfer, as displayed in Fig. 1. The GENius generator (Medtronic, Minneapolis, MN, USA) delivers duty-cycled bipolar and unipolar phased RF energy to all or selected electrode pairs. RF is delivered in a temperature-controlled and power-limited fashion (60 °C, maximum 10 W) with a typical ablation duration of 60 seconds.

The Gold-MEA catheter was introduced into the left atrium via a 10F SL-1 sheath. Using a 0.032 inch guidewire placed in the PV, the catheter was positioned at the antrum of each PV to record local electrical activity at the veno-atrial junction prior to RF energy application, creating PV templates. RF energy was applied using the RF generator (Medtronic GENius, Minneapolis, MN, USA) with a target temperature setting of 60 °C, RF energy setting of 2:1 ratio between bipolar and unipolar energy, and 60-second RF application duration. After each application, PV triggers were assessed. Multiple applications of RF were delivered using the available energy settings until isolation of each PV was achieved. Furthermore, after phased RF ablations were performed at all veno-atrial junctions, the Gold-MEA catheter was used to remap all PV ostia. If the PVs appeared to be incompletely isolated, additional RF applications were delivered using the Gold-MEA until the PV isolation was achieved. No adenosine testing was performed.

To aid in delivering adequate lesions, the RF generator displays temperature and RF power in a green bar when the temperature is >50 °C and RF power >3 W. If either the temperature is <50 °C or the RF power is <3 W, the colour of the bar changes to yellow. This is based on previous research in animal studies which demonstrated that the positive predictive value of an ablation with a temperature >50 °C and RF power >3 W for >30 seconds to predict a transmural lesion was 99 % [16]. In the current study, electrodes with a temperature <50 °C or RF power <3 W were switched off during phased RF energy application.

### Post-ablation management

Patients were hospitalised for at least 24 hours and monitored telemetrically. Oral anticoagulants were resumed immediately after the procedure, with a target INR of 2.5–3.5, in accordance with local guidelines. Low-molecular-weight heparin was administered in a patient-weight dependent dose until the INR was adequate. Complications were defined according to the HRS/EHRA/ECAS expert consensus statement on AF ablation [2]. Cerebral imaging was not performed routinely to assess asymptomatic cerebral embolism.

### Follow-up

A blanking period of 3 months was defined after PVI. Patients visited the outpatient clinic at 3, 6 and 12 months after PVI; this included a 24-hour Holter ECG. All patients were >12 months after their first AF ablation attempt. Patients were immediately referred to the emergency room in case of symptoms. If no arrhythmias could be detected, patients underwent Holter ECG monitoring to exclude arrhythmia recurrence. An attempt was made to discontinue antiarrhythmic drugs in all patients 3 months after the ablation.

### Study endpoints

The primary endpoint of our study was arrhythmia-free survival, defined as patients without AF/atrial flutter/atrial tachycardia recurrence after a blanking period of 3 months. Arrhythmia recurrence was defined as an ECG showing the characteristics of AF/atrial flutter/atrial tachycardia, or on a 30-second telemetry strip, in accordance with the HRS/EHRA/ECAS expert consensus statement on AF ablation [2]. Patients who were still using antiarrhythmic drugs 3 months after the ablation were considered arrhythmia recurrences, in accordance with the HRS/EHRA/ECAS expert consensus statement [2].

### Statistical analyses

Data are mentioned as means ± standard deviation, interquartile range or percentage where appropriate. Statistical significance between variables was calculated by the Student’s t‑test (unpaired) for continuous variables and Chi-square test for categorical variables. Effective electrode-tissue contact creating a transmural lesion was defined as any ablation with a temperature >50 °C and RF power >3 W for over 30 seconds [16]. Patients were categorised into <72 transmural lesions, 72–108 transmural lesions and >108 transmural lesions. Kaplan-Meyer analysis with a log-rank test was used to compare transmural lesion groups. To assess the learning curve, the first 25 patients were categorised to the first cohort and the following 25 patients to the second cohort. The follow-up duration between cohorts was compared with a Mann-Whitney U test. A *p*-value of ≤0.05 was regarded significant. Statistical analysis was performed using IBM Statistics version 22.0 (IBM SPSS Statistics for Windows, 2011: Armonk, New York, USA).

## Results

Fifty consecutive patients with paroxysmal AF were included, mean age was 57.1 ± 11.7 years. No left atrial thrombi were found during the preoperative transoesophageal echocardiogram. Of note, no patients had common left or right PV ostia and no patients had accessory PVs on the pre-ablation CT scan. Baseline characteristics are displayed in Table 1.

### Procedural characteristics

In all 200 PVs, acute PVI was achieved. The mean procedure duration was 111 ± 22 minutes, with a range of 60 to 150 minutes. The mean ablation time was 23.5 ± 6.7 minutes, with a range of 12 to 42 minutes. The mean fluoroscopy time was 20.1 ± 8.1 minutes, with a range of 9 to 45 minutes. None of the patients developed complications related to the procedure and, in particular, no patients developed symptomatic thromboembolic events.

### Ablation outcome

**Fig. 2:**
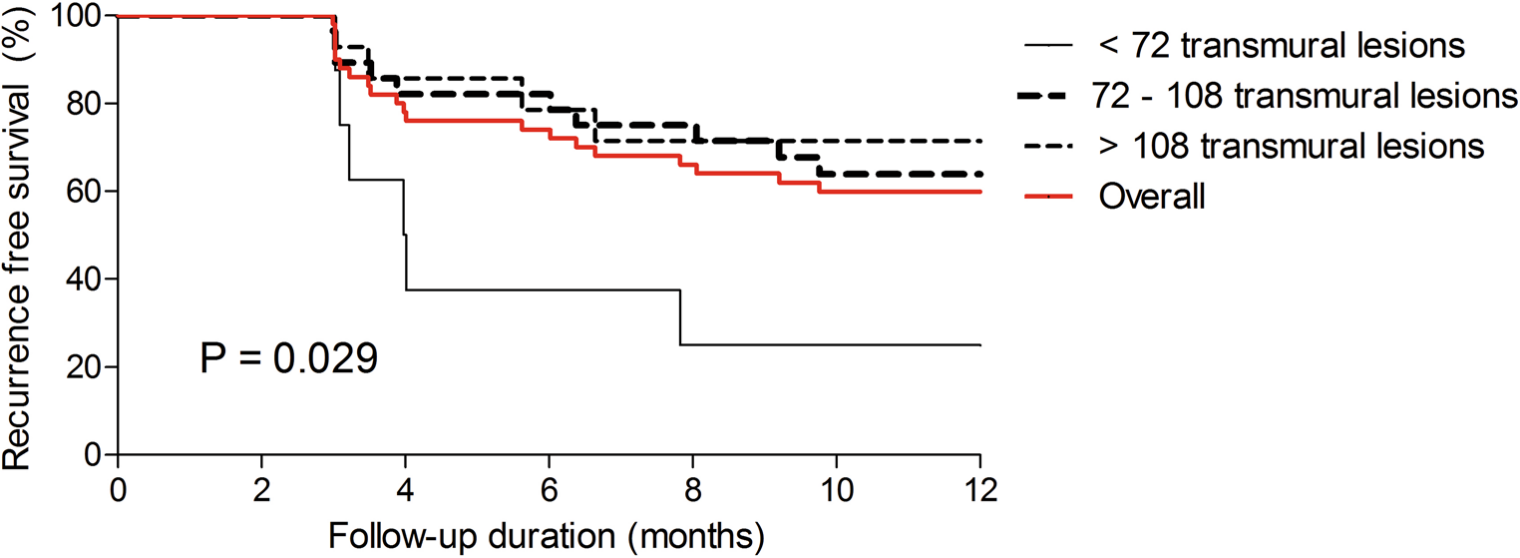
Arrhythmia recurrence-free survival after ablation with the Gold-MEA catheter. This figure displays the AF-free survival after ablation with the Gold-MEA catheter. The overall recurrence free survival is displayed in red. *P*-value between transmural lesion groups. *MEA* multi-electrode ablation

All patients were >1 year after their initial ablation procedure when the present analysis was conducted. One year after ablation, 20 (40 %) of patients developed an arrhythmia recurrence, whereas 30 (60 %) remained free of arrhythmia recurrences and off antiarrhythmic drugs after a single PVI attempt. The arrhythmia-free survival is displayed in Fig. 2.

### Transmural lesions

Transmural lesions, defined as any ablation with a temperature >50 °C and RF power >3 W for over 30 seconds, [16] was achieved in 44.6 % (range 23–62 %) of the total number of lesions delivered. Patients were categorised into: <72 transmural lesions (8 patients); 72–108 transmural lesions (28 patients) and >108 transmural lesions (14 patients). Arrhythmia-free survival after one year was significantly associated with the number of transmural lesions, 25 % in patients with <72 transmural lesions, 64.3 % in patients with 72–108 transmural lesions and 71.4 % in patients with >108 transmural lesions (*p* = 0.029), as displayed in Fig. 2. Of note, none of the patient characteristics were associated with the number of transmural lesions.

### Learning curve

**Fig 3:**
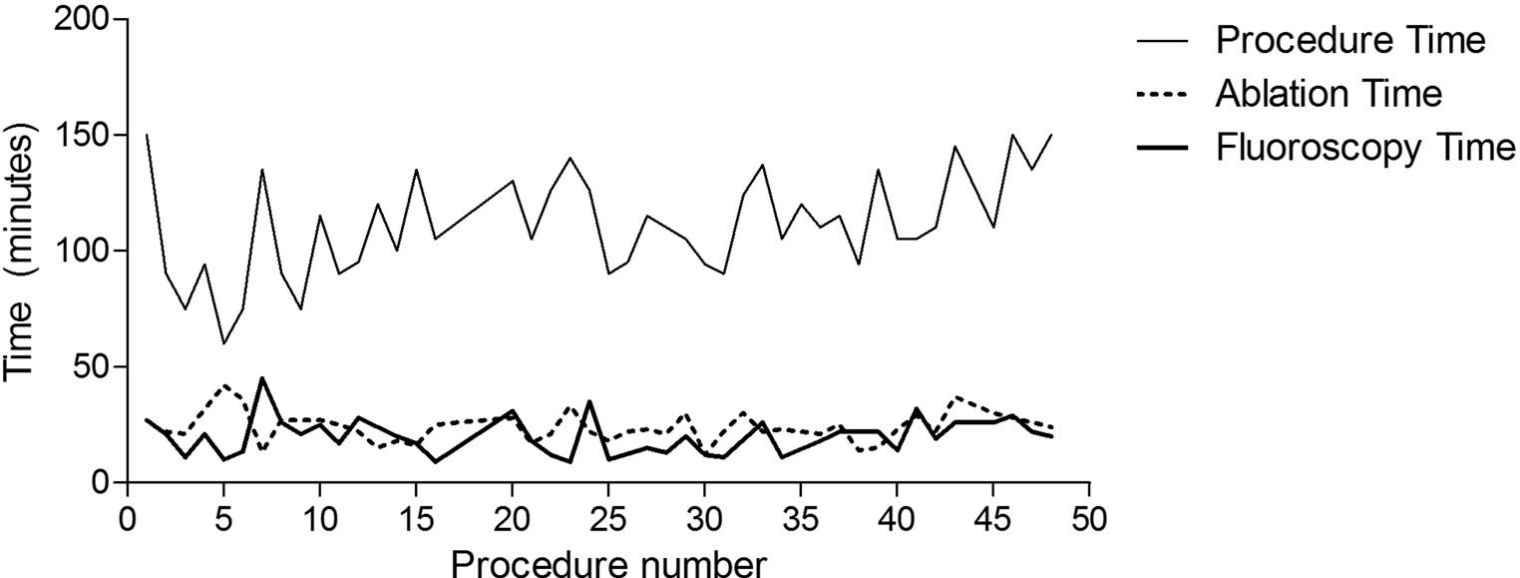
Procedure, ablation and fluoroscopy time. This figure displays the procedure time, ablation time and fluoroscopy time. Note that there appears to be no difference in any of these variables between the first and the second cohort, thus there appears to be no learning curve with the Gold-MEA catheter for operators who are experienced with the Platinum-MEA catheter. *MEA* multi-electrode ablation

The first 25 patients were compared with the following 25 patients in terms of procedural characteristics. As mentioned, acute PVI was achieved in all PVs. Between the cohorts, procedure time (106 ± 25 vs. 116 ± 18 minutes, *p* = 0.11), ablation time (24 ± 7.4 vs. 23 ± 6.0 minutes, *p* = 0.94) and fluoroscopy time (20 ± 9.5 vs. 20 ± 6.4 minutes, *p* = 0.87) were comparable. The number of transmural lesions was comparable in the latter cohort (91 vs. 101, *p* = 0.32). The differences in procedural characteristics are displayed in Table 2 and Fig. 3. In terms of ablation outcome, there was no difference between the two cohorts: of the first 25 patients, 14 (46.7 %) were still arrhythmia free whereas 16 (53.3 %) patients from the second group of 25 patients were still arrhythmia free (*p* = 0.55). Furthermore, there were no significant differences between operators in number of applied transmural lesions (*p* = 0.47) as well as arrhythmia recurrences (*p* = 0.15).

**Table 1:** Baseline characteristics

Patient characteristics	Total (*n* = 50)
Gender female (%)	15 (30 %)
Age (years)	57.1 (±11.7)
BMI (kg/m^2^)	28.3 (±4.2)
Paroxysmal AF	50 (100 %)
AF duration (years)	5.1 (±7.4)
CHADS_2_-VA_2_Sc (range)	1.3 (0–5)
Congestive heart failure	5 (10 %)
LA ventral-dorsal dimension (mm)	41.1 (±3.9)
LVEF (%)	58.8 (±3.2)
History of hypertension	16 (32 %)
History of diabetes mellitus	2 (4 %)
History of TIA/CVA	7 (14 %)

Data are presented as percentages or means ± their SD or ranges where appropriate. *BMI* body mass index, *AF* atrial fibrillation, *LA* left atrium, *LVEF* left ventricular ejection fraction, *TIA* transient ischaemic attack, *CVA* cerebrovascular accident

**Table 2:** Procedural characteristics of the first versus second half of the study cohort

	First cohort (*n* = 25)	Second cohort (*n* = 25)	*P*
Acute isolation (*n*)	100/100 (100 %)	100/100 (100 %)	>0.99
Procedure time (min)	106 ± 25	116 ± 18	0.11
Ablation time (min)	24 ± 7.4	23 ± 6.0	0.94
Fluoroscopy time (min)	20 ± 9.5	20 ± 6.4	0.87
Complications (*n*)	0 (0 %)	0 (0 %)	>0.99
Transmural lesions (*n*)	91 ± 28	101 ± 24	0.32

Data are presented as percentage or means ± their SD where appropriate

## Discussion

The present study reports on the association between transmurality of lesions and AF ablation outcome in Gold-MEA catheter ablation. In conclusion, ablation outcome is poor in case of <72 transmural lesions, but is comparable with other ablation techniques for >108 transmural lesions. Gold-MEA catheter ablation is characterised by a relatively short procedure time, and safety outcome seems to be favourable, although this was a small study.

### Catheter design

The newly designed Gold-MEA catheter builds on its predecessor, but several key improvements have been implemented. First, the new catheter consists of 9 electrodes, to prevent overlap of the 1^st^ and 10^th^ ablation electrode. Second, the ablation electrodes are made of gold instead of platinum. In an animal study with the new Gold-MEA catheter, gold electrodes produced more consistent power delivery than platinum [17] without an increase in microemboli production, potentially due to better passive convective cooling of the electrodes [18]. Last, the catheter system now boasts a 20 forward tilt to improve catheter-wall contact, which improves contact by 56 % according to unpublished data by Medtronic [19].

### Procedure duration

In the present study, we observed that the new Gold-MEA catheter achieved acute PVI in all PVs, with a relatively short procedure time which will result in a more cost-effective approach, since more procedures can be performed in the same amount of time compared with, for example, conventional RF catheter ablation [3, 20] or laser balloon ablation, [6, 21] which are characterised by procedures lasting 2.5–3.5 hours [22].

### Complications

We did not observe any complications with the new Gold-MEA catheter, although this study consists of a limited patient population size. In a previous study performed in our centre, complications after ablation with the Platinum-MEA catheter were only 1.8 %, [3] significantly less than conventional point by point RF catheter ablation. However, as mentioned previously, the Platinum-MEA catheter is no longer in use due to its association with asymptomatic cerebral embolisms. Thromboembolisms were related to overlap between the 1^st^ and 10^th^ electrode in the Platinum-MEA catheter system [23]. This resulted in local overheating of blood and PV tissue, resulting in thromboembolisms [14]. The new Gold-MEA catheter has been re-designed to prevent this local overheating, thereby reducing thromboembolic complications. Furthermore, the Precision Gold Trial [24] will allow assessment of the incidence of asymptomatic cerebral emboli in patients treated with the Gold-MEA catheter. The present study was not aimed at assessing the association between Gold-MEA catheter ablation and asymptomatic cerebral emboli.

### Ablation success

The present study shows that the overall ablation success after a limited follow-up period seemed to be lower compared with other techniques [3, 6, 20, 21]. However, our reported success rate is comparable with the first-generation MEA catheter [25, 26]. Moreover, ablation outcome was clearly associated with the number of transmural lesions. To date, no techniques are available to assess actual lesion depth, and therefore true transmurality cannot be determined. Other AF ablation techniques use surrogates to ascertain if a lesion is transmural. Contact force sensing for conventional RF ablation with a mapping catheter was recently introduced, allowing operators to assess the contact force and force vector [27, 28]. Lesions with a force-time integral >400 gs were significantly associated with more durable PVI lesions and improved ablation outcome [5, 29]. The cryoballoon allows assessment of freezing and thawing temperature, which is indicative of the tissue temperature and thus lesion delivery [30, 31]. Laser balloon ablation allows operators to visually perform lesions that overlap, to ensure transmurality and circumferentiality. Based on experimental data, effective contact is considered a useful marker of transmural lesions for the Gold-MEA catheter. A previous animal study already demonstrated a high positive predictive value of 99 % for effective contact in achieving histologically confirmed acute and chronic transmural lesions [16]. In the present study, we found an association between the number of transmural lesions based on effective contact and ablation outcome. Less than 72 transmural ablations were associated with a significantly poor ablation outcome. Implementation of effective contact into the Gold-MEA catheter ablation may improve ablation outcome. However, this study was not designed to determine the optimal number of transmural lesions to achieve durable PV isolation.

The operators who performed Gold-MEA were experienced with the Platinum-MEA catheter system. No differences between operators were found regarding the number of transmural lesions and arrhythmia recurrences.

However, although lesion quality displayed a significant association with ablation outcome, this was predominantly caused by patients with <72 transmural lesions. The 42 patients with >72 ablation lesions displayed a comparable ablation outcome, even though there was still a substantial variation in the number of transmural lesions and proportion of patients suffering from arrhythmia recurrences. These observations suggest that there are other factors at play that have an impact on ablation success, on top of lesion transmurality. Although the present study was not designed to investigate other factors that impact Gold-MEA ablation results, geometrical variation between patients may be one of these factors. Previous research by our group demonstrated that PV orientation was associated with ablation outcome after multi-electrode ablation [32] and laser balloon ablation, [33] but not after contact force sensing catheter ablation [34]. Future research is necessary to determine if PV orientation may also impact ablation outcome after Gold-MEA ablation.

## Limitations

The present study consists of a limited patient sample size, and therefore claims on safety and PVI success may be overestimated. Future research is warranted including more patients with a more extended follow-up period. Single procedure ablation success is reported, whereas most previous studies reported success after multiple PVI attempts. No adenosine testing was performed to assess for dormant conduction of PVs, which could potentially result in a higher success rate.

## Conclusion

AF ablation can be performed successfully with the Gold-MEA catheter, with a favourable safety profile. A higher number of effective lesions, as a surrogate marker for transmurality of lesions, was associated with freedom of AF during follow-up.
